# Wearable Photomedicine for Neonatal Jaundice Treatment Using Blue Organic Light‐Emitting Diodes (OLEDs): Toward Textile‐Based Wearable Phototherapeutics

**DOI:** 10.1002/advs.202204622

**Published:** 2022-10-30

**Authors:** Seungyeop Choi, Yongmin Jeon, Jeong Hyun Kwon, Chunhwa Ihm, Seung Yeon Kim, Kyung Cheol Choi

**Affiliations:** ^1^ School of Electrical Engineering Korea Advanced Institute of Science and Technology (KAIST) Daejeon 34141 Republic of Korea; ^2^ Department of Biomedical Engineering Gachon University Seongnam 13120 Republic of Korea; ^3^ Department of Display and Semiconductor Engineering SUN MOON University Choongcheongnam‐do Asan 31460 Republic of Korea; ^4^ Department of Laboratory Medicine Daejeon Eulji Medical Center Eulji University School of Medicine Daejeon 35233 Republic of Korea; ^5^ Department of Pediatrics Nowon Eulji Medical Center Eulji University School of Medicine Seoul 01830 Republic of Korea

**Keywords:** bilirubin, neonatal jaundice, organic light‐emitting diodes (OLEDs), photomedicine, textile, wearable electronic devices

## Abstract

Neonatal jaundice is a very common disease in newborns and can lead to brain damage or death in severe cases. Phototherapy with light‐emitting diode (LED) arrays is widely used as the easiest and fastest way to relieve jaundice in newborns, but it has distinct disadvantages such as loss of water in the patient, damage to the retina, and separation from parents. In this paper, a novel light source‐based phototherapy for neonatal jaundice is proposed using a textile‐based wearable organic light‐emitting diode (OLED) platform that can move flexibly and conform to the curvature of the human body. The soft and flexible textile‐based blue OLED platform is designed to have a peak wavelength of 470 nm, suitable for jaundice treatment, and shows performance (>20 µW cm^−2^ nm^−^^1^) suitable for intensive jaundice treatment even at low voltage (<4.0 V). The textile‐based OLEDs fabricated in this study exhibit an operating reliability of over 100 h and low‐temperature operation (<35 °C). The results of an in vitro jaundice treatment test using a large‐area blue OLED confirm that the bilirubin level decreases to 12 mg dL^−1^ with 3 h of OLED irradiation.

## Graphical abstract

Kyung Cheol Choi et al. develops a novel light source‐based photomedical approach for neonatal jaundice treatment, using a textile‐based wearable organic light‐emitting diode (OLED) platform that can move flexibly and conform to the curvature of the human body. The effectiveness of the blue OLED jaundice treatment is verified by in vitro test, and effective and uniform treatment performance is confirmed. 

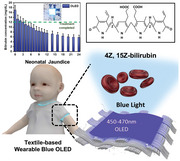

## 1 Introduction

Among the growing number of wearable electronic devices being developed, researchers are actively pursuing biomedical applications.^[^ ^1^, ^2^, ^3^ ^]^ Wearable electronics‐based medical devices have the advantage of being able to both diagnose health conditions and treat diseases without the usual constraints of time and space. In particular, light‐based wearable biomedical applications, which are very safe and effective because of their non‐invasive characteristics, are being studied for various sensors and photomedicine applications including pulse oximeters, phototherapeutics, and photodynamic therapeutics.^[^ ^4^, ^5^, ^6^ ^]^

Phototherapy can treat diseases using low‐power light, and is being investigated for jaundice treating, wound healing, wrinkle improvement, pain relief, and inflammation relief, using various treatment principles.^[^ ^7^, ^8^, ^9^ ^]^ In particular, photomedicine is very effective for the treatment of pathologic jaundice in newborns and is being widely used in hospitals. Bilirubin, a toxic substance that causes jaundice, is produced when iron‐containing proteins such as hemoglobin are broken down. Bilirubin is normally detoxified by the liver and excreted in bile. Newborns have red blood cells with a short lifespan, but their liver metabolism is immature, so they produce a lot of bilirubin.^[^ ^8^ ^]^ Pathological jaundice, which can develop between 24 h and 2 weeks after birth, must be treated immediately, otherwise it can cause hearing impairment or develop into nuclear jaundice, which can lead to cerebral palsy or death.^[^ ^10^, ^11^, ^12^, ^13^, ^14^, ^15^ ^]^

In newborns, pathological jaundice treatment is carried out using various methods including phototherapy, drug administration, and exchange transfusion.^[^ ^16^, ^17^, ^18^ ^]^ Phototherapy with blue light wavelengths of 450 to 490 nm have been used in clinical practice for neonatal hyperbilirubinemia for ≈60 years.^[^ ^19^ ^]^ Phototherapy involves exposing the bilirubin that has accumulated in the body to light, transforming it into an isomer and releasing it, bypassing the liver metabolism.^[^ ^20^ ^]^ This treatment improves symptoms by reducing the concentration of bilirubin in the body. In addition, as most neonatal jaundice can be treated with phototherapy, it is currently the most widely used intervention. When using this phototherapy method for neonatal jaundice, bilirubin can be excreted from the body after optical isomers are formed by blue light^[^ ^20^ ^]^.

Initially, treatment was performed using a fluorescent lamp or a cold cathode fluorescent lamp (CCFL) light source.^[^ ^21^, ^22^ ^]^ After additional research, the most effective wavelength of light was determined to be 450 to 490 nm, and blue light‐centered treatment is now becoming the mainstream approach.^[^ ^8^ ^]^ Recently, because of problems with environmental pollution, toxicity to the human body, operating lifetime, and the size of the light source, and fluorescent lamps used in industries and homes are being replaced by light‐emitting diodes (LEDs). However, these LEDs are mainly being used as an installation platform because of their specific characteristics, being a point light source, rigid, bulky, and hot, and accordingly, their application as a wearable light source has been limited.^[^ ^23^, ^24^ ^]^

In hospitals, when the concentration of bilirubin in the blood of a newborn exceeds therapeutic range, the newborn is hospitalized in the neonatal intensive care unit (NICU), placed in an incubator, and treated with light from LEDs mounted on a stand.^[^ ^8^, ^25^, ^26^, ^27^ ^]^ Although this method is very effective at relieving the symptoms of neonatal jaundice, it is necessary to isolate the newborns from their parents and stop breastfeeding for the duration of treatment. In addition, as the baby's body must be exposed in the incubator, strong blue light causes retinal damage, so the newborn's eyes must be completely covered with a blindfold.^[^ ^28^ ^]^

To address these problems, research is being conducted on novel light sources, including a wearable type platform that can be worn by newborns. Recently, various platforms that can be worn or attached have been investigated, using light sources such as OLED (organic light‐emitting didoes) and QLED (quantum‐dot light‐emitting didoes) based on thin film and surface light sources.^[^ ^5^, ^29^, ^30^ ^]^ In addition to applications in photomedicine such as wound healing and collagen regeneration using these novel light sources, research is being actively conducted on sensors such as pulse oximeters.^[^ ^31^, ^32^, ^33^, ^34^, ^35^ ^]^

In this study, a potential new type of wearable phototherapy medical device was demonstrated, using a textile‐based blue OLED. To study its performance and reliability, and suitability for jaundice treatment, a textile‐based wearable blue OLED was designed and manufactured, and the jaundice treatment effect was confirmed using the blue OLED. Thanks to the characteristics of the OLED surface light source, it can be closely attached to the human body. Its superior treatment performance and uniformity, compared to conventional LEDs, were confirmed through experiments. These textile‐based wearable OLEDs are very thin and light, and flexibly deform to follow the curves of the human body. The feasibility of their use in a wearable form for the treatment of jaundice in newborns was confirmed.

## 2 Results

### 2.1 Fabrication of Blue‐Wavelength OLEDs for Neonatal Jaundice

As the light source used to treat neonatal jaundice has changed from fluorescent lamps to LEDs, studies have been conducted to evaluate treatment efficacy based on the wavelength of each light source. Many research reports have verified the effectiveness of LED treatment.^[^ ^21^, ^22^, ^36^ ^]^ However, further studies are required to investigate the structural design and manufacturing method for applying OLEDs, a next‐generation light source, to neonatal jaundice and to validate the efficacy of the jaundice treatment.

Typically, when a newborn is lying in an incubator, light treatment efficiency is low because the infant's back does not receive light. The use of clothing with LEDs as the light source has been reported for neonatal jaundice phototherapy, with a purpose similar to this study.^[^ ^36^, ^37^ ^]^ This is a meaningful study in that it suggests the possibility of treating jaundice with LEDs in the form of a wearable platform that can be worn by humans. However, that proposed LED treatment was not in a form that children can wear, and its practicality was very low, so it has not received much attention. Specifically, the rigid LED array method is disadvantageous in terms of wearability, and there may also be a problem of local heating because it irradiates high‐energy light locally for treatment.

**Figure 1:**
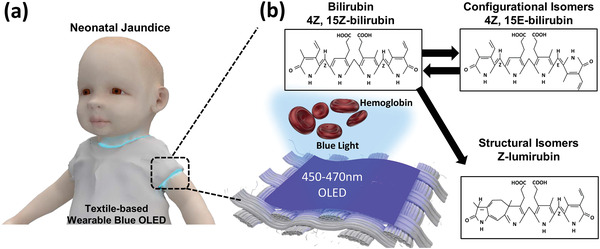
Concept of the textile‐based wearable blue OLED‐based neonatal jaundice phototherapy: a) Schematic illustration of a textile‐based wearable jaundice treatment platform. b) Mechanism of treatment of jaundice through bilirubin isomer formation by textile‐based blue OLED and subsequent excretion.

As an alternative, a textile‐based wearable blue OLED is proposed in this study that will ultimately allow the treatment of jaundice in the form of flexible clothing, as shown in the conceptual image in **Figure** **1**a This approach solves all the problems that typically arise when using conventional light sources for the treatment of jaundice. In addition to developing a process for manufacturing a flexible textile‐based blue OLED, further optimization was required to realize an OLED that emitted light in the 450–490 nm band, which is the most effective for neonatal jaundice phototherapy. Using the proposed textile‐based blue OLED, it was possible to induce the formation of isomers from bilirubin that could be excreted from the body, even when it was worn in the form of clothing (Figure 1b).

When blue light is applied to bilirubin, 4Z,15E‐bilirubin isomers and Z‐lumirubin isomers are formed. The 4Z,15E‐bilirubin isomer formed after phototherapy accounts for ≈20% of the total bilirubin. However, as its serum half‐life is very long, more than 10 h, the effect of phototherapy is not great.^[^ ^36^ ^]^ On the other hand, although the Z‐lumirubin isomer is only ≈5% of the total; it has a half‐life of less than 2 h and is irreversible without change, so it can be efficiently excreted. Consequently, the phototherapeutic effect of jaundice is mainly due to Z‐ lumirubin.^[^ ^36^ ^]^

Before manufacturing the textile‐based blue OLED platform, it was necessary to design an effective blue OLED for the treatment of jaundice. Details on the fabrication and evaluation of the OLEDs designed for the treatment of jaundice are described in the Experimental Section.

In this study, the DSA‐Ph (1‐4‐Di‐[4‐(*N*, *N*‐diphenyl)amino]styryl‐benzene) emission phosphor, which has excellent operational reliability, was selected to ensure a stable phototherapy application. The photoluminescence of the DSA‐Ph used as the emission layer (EML) dopant is broad wavelength, with two peaks at 470 and 500 nm, as shown in Figure S1, Supporting Information. However, as the blue OLED used for jaundice treatment requires a wavelength of 450 to 490 nm, it is necessary to control and amplify the wavelength through optical design.

**Figure 2:**
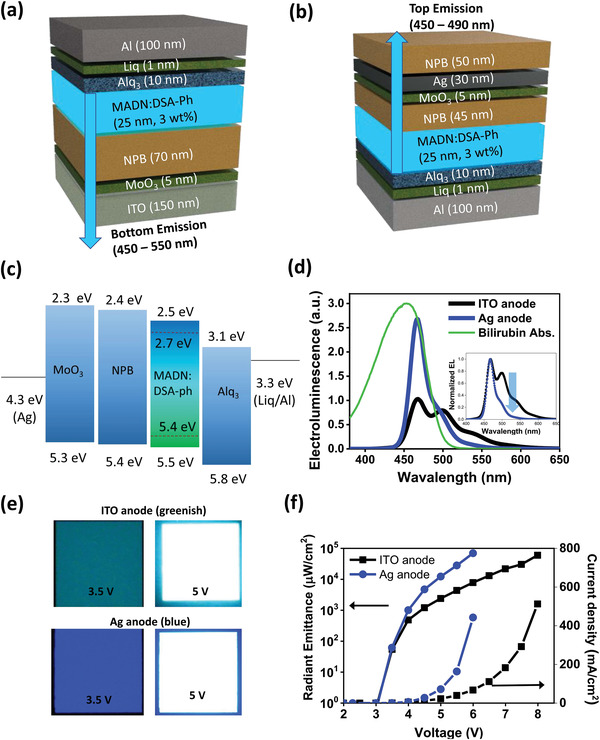
Design and manufacture of a blue OLED for the treatment of jaundice: a) The reference blue OLED structure, based on a general ITO anode (bottom emission). b) Microcavity blue OLED structure to produce the appropriate wavelength and output for jaundice treatment (top emission). c) Energy level diagram according to the material and structure of the blue OLED. d) Absorption spectrum of bilirubin and the emission spectrum of the reference OLED and microcavity OLED. e) Emission images of the reference OLED and microcavity OLED. f) Radiant emittance and current density graph according to OLED driving voltage.

The standard blue OLED is a bottom emission type, based on a transparent ITO (indium tin oxide) anode, as shown in **Figure** **2**a. To realize a textile‐based wearable blue OLED platform, it is necessary to design a top emission structure, where light is extracted in the direction opposite to the opaque textile. As shown in Figure S2, Supporting Information, the material and electrode thickness were optimized for resonance near 470 nm using optical simulations, based on two‐beam interference and Fabry–Perot interference. The simulation indicated that a strong microcavity effect can be generated between a semi‐transparent Ag electrode and a highly reflective Al electrode by replacing the ITO electrode with Ag (30 nm), as shown in Figure 2b. As the microcavity wavelength is determined by the thickness of the inner organic material, the thickness of the inner organic material layer was adjusted, considering the electron–hole balance. In addition to the optical design, it was necessary to design the OLED structure considering the energy level, so that excitons could be electrically well formed. Therefore, as shown in Figure 2c, each layer of the blue OLED was designed by considering the work function and energy level, so that the electrons and holes could be transferred well.

To effectively form bilirubin isomers using the OLED and to discharge them outside the body, it is necessary to maximize the light in the wavelength region absorbed by bilirubin. As shown in Figure 2d, the peak absorption wavelength of bilirubin is 450–470 nm.^[^ ^38^, ^39^ ^]^ The top emission blue OLED manufactured with the microcavity design emitted light suitable for this bilirubin absorption wavelength, in contrast to the previous ITO‐based reference blue OLED. This made it possible to effectively strengthen the wavelength of the blue light and increase the light output of the wavelength band for effective treatment by more than 100% compared to the reference.

As shown in Figure 2e, the ITO‐based reference OLED has green–blue emission characteristics, and the wavelength‐optimized OLED with the Ag‐based microcavity effect shows blue emission characteristics. The blue OLED manufactured with this optical and structural design not only provided effective wavelengths for the treatment of jaundice but also produced a high radiant emittance of 10^5^ µW cm^−2^ even at a low voltage of 6 V or less (Figure 2f). As shown in Figure S3, Supporting Information, in terms of luminance, it also showed high performance, of over 10^4^ cd m^−2^ at 6 V.

### 2.2 Textile‐Based Wearable Blue OLED for Neonatal Jaundice

**Figure 3:**
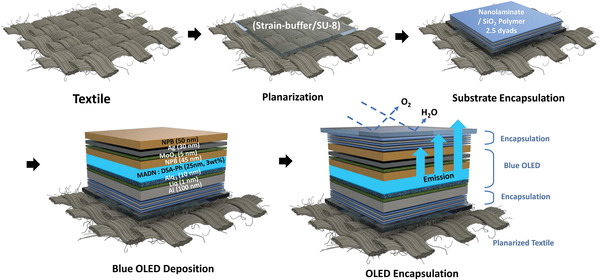
Manufacturing process of the textile‐based blue OLED platform for jaundice treatment.

To achieve a wearable OLED‐based with the best performance for jaundice treatment, the designed blue OLED was fabricated on a textile substrate, and then underwent a passivation process for reliability. As OLEDs are typically manufactured using a thin film process with nm‐scale thickness, they can only be stably manufactured on a substrate that has a surface roughness in the nm‐scale. As textile substrates generally have a very high roughness, a process for planarizing the textile substrate is required. Our group established a textile planarization process in a previous study, and it was used to fabricate the wearable blue OLED platform in the current study.^[^ ^40^, ^41^, ^42^ ^]^ As shown in **Figure** **3**, using the strain‐buffer/SU‐8 a planarization process, a nanometer‐scale planarization substrate can be obtained while retaining the flexibility of the textile.

A subsequent encapsulation process is absolutely necessary because OLEDs are vulnerable to moisture and oxygen. Therefore, before the OLED was fabricated, a process was carried out to laminate 2.5 dyads each of SiO_2_ polymer and a nanolaminate composed of Al_2_O_3_ and ZnO on a planarized textile substrate.^[^ ^43^ ^]^ Afterward, blue OLEDs optimized for the treatment of jaundice were sequentially fabricated through thermal deposition, followed by encapsulation with a 2.5 dyads structure. In addition, an adhesive PET transfer process was performed to increase the washing reliability of the OLED and to ensure that only the textile and pure PET parts were in contact with the skin. Like a previous textile‐based LED blanket in wide use,^[^ ^44^ ^]^ it is expected that the textile‐based wearable OLED fabricated with this process can also be applied to the human body without side effects. In fact, OLEDs are currently being used without ill effect in various wearable biomedical applications, in the form of patches attached to the human body.^[^ ^33^, ^35^ ^]^

**Figure 4:**
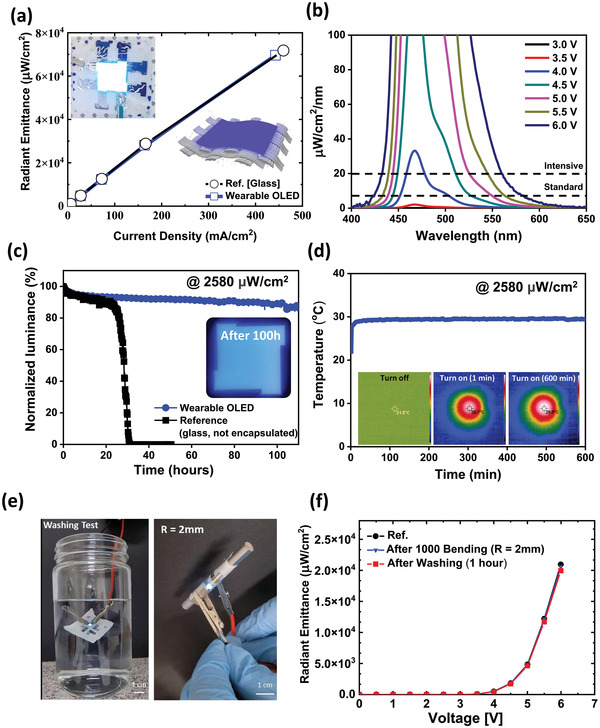
Evaluation of operating performance and reliability of the textile‐based OLED platform: a) Comparison of radiant emittance performance according to current density of the textile‐based OLED and glass‐based OLED. b) Output intensity of wearable OLED according to voltage and wavelength. c) Wearable OLED operational lifetime test. d) Wearable OLED operational temperature test. e) Wearable OLED with flexibility and cleaning reliability. f) Comparison of the wearable OLED device characteristics before and after bending and washing tests.

It was then necessary to analyze whether the textile‐based blue OLED platform manufactured using this process had the proper performance and reliability for actual jaundice treatment. The textile‐based blue OLED for the treatment of jaundice fabricated by this process produced a stable surface light source, as shown in the picture in **Figure** **4**a and showed almost the same high performance as the blue OLED fabricated on a glass substrate. Neonatal jaundice treatment is effective in the 430–490 nm band, and as mentioned above, it is known that the treatment effect is highest in the 450–490 nm band. At this time, the standard intensity required for phototherapy is 10 µW^−1^ cm^−2^ nm^−1^ or more. For intensive treatment, the patient may be irradiated with light at the level of 20 µW^−1^ cm^−2^ nm^−1^ by increasing the intensity.^[^ ^8^ ^]^ With clothing‐type OLEDs, unlike standard‐type LEDs, the distance between the patient and the light source is very close, so the intensity of the light source becomes the intensity of the light irradiated to the patient.

As shown in Figure S4, Supporting Information,the optimized textile‐based blue OLEDs emitted an intensity of ≈2000 µW^−1^ cm^−2^ nm^−1^ even at a low voltage of 6 V, confirming that there was no shortage of light output. In addition, it was confirmed that the standard and intensive light intensity applied to general jaundice treatment was satisfactory at 4.0 V (Figure 4b). **Table** **1** shows the key electro‐optical parameters according to the driving voltage of the textile‐based blue OLED.

**Table 1:** Key parameters of the textile‐based blue OLED

Voltage [V]	Current density [mA cm^−2^]	Luminance [cd m^−2^]	Total optical power [µW cm^−2^]	Therapeutic power (425–490 nm) [µW cm^−2^]
3.0	0.01	0.51	1.36	1.09467
3.5	0.12	43.08	129.67	108.5804
4.0	0.97	498.47	1516.60	1273.419
4.5	3.38	1783.70	5446.76	4577.659
5.0	8.09	4236.20	13 002.42	10 939.51
5.5	18.14	9206.90	28 468.20	24 001.83
6.0	49.60	21 482.00	67 345.72	56 970.89

Before employing the textile‐based blue OLED platform to treat jaundice, it is essential to achieve not only output performance but also operating stability As shown in Figure 4c, the wearable OLED platform took ≈100 h to reach LT90 (90% of initial power) with an initial power of ≈2580 µW cm^−2^ (intensive treatment level). The textile‐based OLEDs prepared with 2.5 dyads thin film encapsulation demonstrated flexibility as well as reliable performance when exposed to oxygen and moisture. Specifically, the results confirmed the textile‐based encapsulated OLEDs have a longer operational lifetime than glass OLEDs without encapsulation. As it usually takes several hours to treat jaundice, this ensures sufficient operating reliability.

In addition, as the textile‐based OLEDs light source are in the form of clothing that a person must wear, it is necessary to determine whether there is a problem with heat generation. As shown in Figure 4d, temperature changes induced by the textile‐based blue OLEDs were observed using a thermal imaging camera. The operating temperature remained at ≈29.5 °C despite long operation, for 10 h. Considering that low‐temperature burns can occur at temperatures above 42 °C, the wearable OLED platform was judged to have a very stable operating temperature, and a level applicable to the human body.

**Figure 5:**
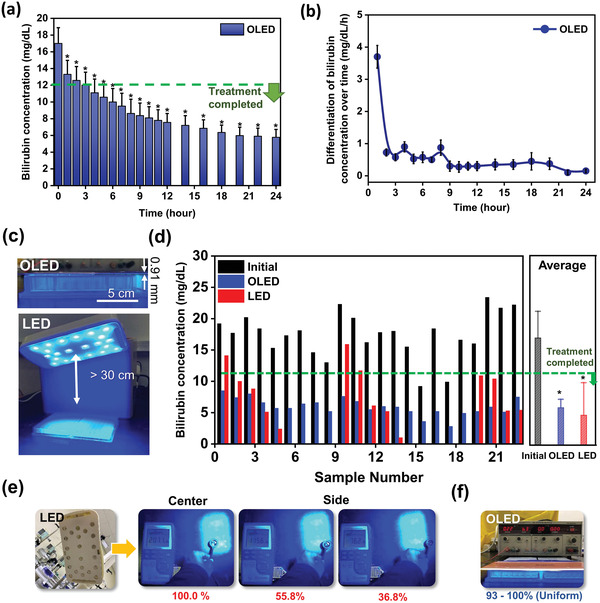
Comparison of in vitro bilirubin phototherapy effects according to the application of the blue OLED light source (The bilirubin concentration data are presented as mean ± standard deviation [n = 23]). In addition, * indicates that the *p*‐value is less than 0.001 : a) Graph showing the reduction in bilirubin concentration according to blue OLED irradiation time. b) Differentiation of bilirubin concentration by blue OLED irradiation time. c) Comparison of irradiation distance for the surface light source OLED and the point light source LED. d) Comparison of bilirubin reduction in neonatal serum when irradiated by the OLED and LED light sources, respectively. e) Non‐uniform characteristics of the point light source LED array with long‐distance irradiation. f) Uniform characteristics of the surface light source OLED at close irradiation.

In order to confirm the practicality of the textile‐based OLED, a flexibility test was conducted with the wearable OLED. Textile substrates are known to be very flexible and generally have a low Young's modulus due to their woven structure.^[^ ^42^ ^]^ Textile‐based OLEDs can be employed for clothes when they have reliability in the mm‐scale bending radius. A wearable OLED was subjected to a bending test 1000 times at a bending radius of 2 mm, and the performance of the OLED remained almost the same (**Figure** **5**e,f). In addition, as wearable OLEDs are manufactured using textile substrates, reliability after washing is also important. Our group previously reported an encapsulation technique using a SiO_2_‐based polymer that is reliable for water washing, and the same encapsulation was applied in this wearable OLED study.^[^ ^43^ ^]^ When a water washing test was conducted at 200 rpm for 1 h using a textile‐based OLED, the measured device performance was almost the same as the unwashed OLED (Figure 5e,f). These results further confirm that the textile‐based blue OLED platform has sufficient performance and reliability for jaundice treatment.

### 2.3 Verification of OLEDs’ Efficacy for Neonatal Jaundice

It was confirmed that the textile‐based OLED had sufficient electro‐optical properties and reliability for the neonatal jaundice phototherapy application. A further study was conducted to confirm the effectiveness of the OLEDs with these characteristics for reducing the concentration of bilirubin in the blood. This experiment was approved by the Institutional Review Board (IRB), and an in vitro experiment was conducted in which blood from a newborn with jaundice caused by hyperbilirubinemia was collected, centrifuged, and then irradiated with light. Blood was collected from a total of 23 neonates. In the case of whole blood, as red blood cells are destroyed and the bilirubin concentration continuously increases, the experiment was conducted using serum and bilirubin isolated from red blood cells. In this experiment, a large‐area 8.1‐in. blue OLED (LG Display Co. Ltd) was used to clearly confirm the effect of the OLED surface light source for neonatal jaundice treatment, compared to the previous stand‐type LED. The emission size of this OLED was 189 mm in length, 39 mm in width, and 0.91 mm in thickness, which are sufficient to provide a uniform OLED light source in close contact with a 96‐well plate. This experiment was designed to analyze the advantages of the jaundice treatment effect using a uniform large OLED surface light source compared to the conventional LED point light source. The emission peak was 460–470 nm and the experiment was performed at ≈10 µW cm^−2^ nm^−^^1^, which corresponds to the standard power of jaundice treatment. As the shape of the emission wavelength of this large‐area blue OLED was similar to that of the textile‐based blue OLED platform, a similar treatment effect could be expected (Figure S5, Supporting Information).

The blue OLED irradiated light directly above the serum, similar to how a light treatment platform adhered to the human body in the form of clothing would operate. Figure 5a shows the change in bilirubin concentration with time after irradiating the newborn serum with light using this blue OLED. It took ≈3 h to reach a bilirubin concentration of 12 mg dL^−1^, which was the point at which the treatment was completed. In other words, the results of the in vitro experiments confirmed that a sufficiently effective bilirubin treatment was possible using the blue OLED light source. In addition, it showed that the decrease in bilirubin concentration was greatest within the initial 3 h of blue OLED irradiation (Figure 5b). In other words, by immediately lowering the level of bilirubin in the blood of a newborn, the textile‐based blue OLED platform could be used to prevent or treat jaundice even when it was difficult for a newborn to go to a hospital or hospitalization was difficult.

The advantages of the surface light source OLED were analyzed and compared with the previous stand‐type LED. For this comparison, a stand‐type blue LED light therapy device (Atom Co., Ltd, BILI‐THERAPY Spot Type) used in an actual hospital NICU was also tested with the same serum (Figure 5c). For the stand‐type blue LED light therapy device used, shown in Figure 5c, 22 LEDs with peak wavelengths of 460–470 nm were included in one device, and the output intensity was 20 µW cm^−2^ nm^−^^1^. The full width of half maximum (FWHM) of the LED was ≈30 nm, and it was confirmed that the OLED also had a FWHM of ≈30 nm when driven at 6 V. It is known that LEDs have a very narrow FWHM, and as the OLED in this study had a structure that maximizes light extraction at a specific wavelength due to the microcavity effect, it was confirmed that the FWHM was very narrow, like the LED.

A blue LED was used as a point light source array to irradiate the serum with light at a distance of ≈30 cm, the same distance as the current treatment. As an OLED is a surface light source, light can be uniformly irradiated over a wide range without generating heat. However, when an LED, which is a point light source, is placed close to a sample, light is irradiated only to a very local area and there is a resulting heat effect.

While OLEDs and LEDs have similar luminescence mechanisms, they are completely different light sources with distinct differences in appearance and material properties. Therefore, the experiment was conducted considering the particular characteristics of each light source, and the experimental conditions were set to appropriately accomodate the characteristics of each light source.

Figure 5d shows the experimental results, which show observed changes in bilirubin levels using OLEDs and LEDs. In general, when the bilirubin concentration fell below 12 mg dL^−1^, it was considered to have had sufficient treatment, and phototherapy was stopped and natural healing induced.^[^ ^8^, ^25^, ^26^, ^27^ ^]^ The average initial bilirubin concentration was ≈16.9 mg dL^−1^, and it was confirmed that the bilirubin concentration decreased to an average of 5.9 mg dL^−1^ in the OLEDs experimental group and 4.7 mg dL^−1^ in the LEDs experimental group after 24 h of irradiation (Figure 5d). As the light output of the LEDs is much higher than that of OLEDs, it was a natural result that bilirubin concentration would decrease more, on average, with the LEDs.

It was observed that in the OLEDs’ experimental group, the bilirubin concentration decreased very uniformly, while the bilirubin concentration decreased very unevenly in the LEDs’ experimental group (Figure 5d). When the light intensity of the LEDs was measured with a laser power meter at a distance of 20 cm, it was confirmed that the intensity of the output varied greatly, even with a small change in position (Figure 5e). Accordingly, when the averages were compared, while the effect of the LEDs seemed to be better, the bilirubin concentration of some samples did not meet the treatment standard (12 mg dL^−1^), and the bilirubin concentration was found to be excessively decreased (0 mg dL^−1^) in others. In the center position, the bilirubin levels were excessively reduced to zero levels because the LED intensity (20 µW cm^−2^ nm^−1^) there was locally greater than the OLED intensity (10 µW cm−^2^ nm^−1^). However, in the side position of the LED, the intensity was lowered to 36.8% of that of the center, and the bilirubin concentration there was higher than that of OLED because it was irradiated with a lower intensity.

As bilirubin has a dual behavior depending on the concentration, it is important to maintain an appropriate level.^[^ ^45^ ^]^ High bilirubin levels above 12 mg dL^−1^ after LED treatment may not completely cure jaundice in newborns and may lead to a dangerous situation.^[^ ^8^, ^25^, ^26^, ^27^ ^]^ In addition, because moderate levels of bilirubin act as antioxidants, excessively lowering the bilirubin concentration to the 0 mg dL^−1^ level can also have a negative effect. As the LED light source‐based jaundice treatment has limitations in terms of uniformity, in some cases, treatment may be less or side effects may occur. However, as the OLED is in the form of a surface light source, as shown in Figure 5f, there is no heat problem, so it can be adhered to the skin. When the 9‐point uniformity of each emitting area of the OLED was measured, it showed very uniform characteristics (Figure S6, Supporting Information). Therefore, as shown in Figure 5d, all 23 samples exhibited a uniform level of reduced bilirubin concentration.

In this study, a textile‐based blue OLED with the wavelength, radiant emittance, and reliability required for neonatal jaundice treatment was demonstrated. In addition, the neonatal jaundice treatment effect was confirmed through an in vitro test using an OLED surface light source, and the advantages of the treatment effect compared to LEDs were analyzed. The uniform OLED surface light source in this study not only showed effective jaundice treatment, corresponding to the LED device currently used in hospitals, but also showed superior uniform treatment performance. Additional preclinical and clinical trial evaluations of the neonatal clothing‐based textile OLED jaundice treatment are required next, and it is expected that wearable photomedicine using textile OLEDs will be possible in the future.

## 3 Conclusion

In summary, this study confirmed the potential for treating neonatal jaundice with a wearable device, using a textile‐based wearable blue OLED platform. To optimize the blue OLED for jaundice treatment, a high‐performance blue OLED with a peak near 470 nm was manufactured with a top emission structure and microcavity optical design. In addition, we succeeded in manufacturing a blue OLED on a real textile substrate to produce a wearable platform that can be worn by people. This platform demonstrated the ability to treat intensive jaundice by achieving a radiant emittance intensity of ≈2000 µW cm^−2^ even at a voltage as low as 4.5 V. This wearable OLED platform also exhibited a low operating temperature of ≈30 °C, too low to cause burns on the human body. It also showed reliable operation for more than 100 h.

The blue OLED with a peak wavelength of ≈460–470 nm took 3 h to lower the bilirubin concentration to 12 mg dL^−1^ or less at the standard jaundice treatment intensity (10 uW cm^−2^ nm^−^^1^), confirming the potential for real jaundice treatment. In addition, the surface light source OLED, which can be closely attached to the human body, has the advantage of very uniformly reducing the concentration of bilirubin in the treatment area, compared to a point light source LED array, which requires treatment at a certain distance. In conclusion, tests with the textile‐based wearable blue OLED platform in this study confirmed the possibility that neonatal phototherapy clothing can provide a high‐quality treatment environment for many parents who need to monitor their children's health.

## 4 Experimental Section

### Blue OLED Fabrication

In this experiment, the OLED was fabricated using a thermal evaporator (D.O.V co., ltd). Each organic/inorganic material was deposited on a glass or fiber substrate with a size of 25 mm × 25 mm under vacuum conditions of 10^−6^ torr. The bottom emission blue reference OLED structure was fabricated in the following order: first, molybdenum trioxide (MoO3, 5 nm) was deposited as a hole injection layer (HIL) on ITO, as an anode. Subsequently, after *N*, *N*′‐di(1‐naphthyl)‐*N*, *N*′‐diphenyl‐(1,1′‐biphenyl)‐4,4′‐diamine (NPB, 70 nm) as a hole transport layer (HTL) was deposited, 2‐methyl‐9,10‐di(2‐naphthyl)anthracene (MADN) : p‐bis(p‐*N*,*N*‐di‐phenyl‐aminostyryl) (DSA‐Ph) (Host: Dopant) (25 nm, 3 wt%) as an Emission Layer (EML) was deposited. Finally after, Tris(8‐hydroxyquinolinato)aluminium (Alq_3_, 10 nm) was deposited as the Electron Transport Layer (ETL), 8‐Quinolinolato Lithium (Liq, 1 nm) as Electron Injection Layer (EIL), and aluminum (Al, 100 nm) as the cathode.

As the type of microcavity OLED is top emission, it was fabricated in the reverse order of the reference OLED: After the cathode (Al, 100 nm) and EIL (Liq, 1 nm) were deposited with the same material and thickness, ETL Alq_3_ (10 nm) was deposited. EML was also deposited with the same recipe (MADN : DSA‐Ph, 3 wt%), and HTL (NPB, 45 nm) was subsequently deposited. After HIL (MoO_3_, 5 nm) was deposited, Ag (30 nm) was deposited on the anode to induce a microcavity. Finally, an additional 50 nm of NPB was deposited as an optical capping layer.

### Barrier Fabrication

Atomic layer deposition (ALD) and a spin coater were used as barriers to fabricate the textile‐based blue OLED platform in this study. ALD was performed at a chamber temperature of 70 °C and a vacuum degree of 3.0 × 10^−1^ torr, and a trimethylaluminum (TMA) source was used to deposit Al_2_O_3_, and a dimethylzinc (DEZ) source was used to deposit ZnO, respectively. A 30 nm nanolaminate oxide layer composed of five pairs of Al_2_O_3_ (3 nm) and ZnO (3 nm) was fabricated. It was accelerated on a spin coater for 30 s to reach 5000 rpm, maintained for 3 s, and then cured on a 70 °C hot plate for 20 min to create a 300 nm SiO_2_ polymer film. Both the nanolaminate oxide layer and the SiO_2_ polymer layer were fabricated in three dyads structure by repeating the process three times.

### Device Characterization

The electrical characteristics of the blue OLED in this study were measured with a source‐meter (Keithley 2400, Keithley Co., Ltd), and the optical characteristics of the blue OLED light source were measured using a spectro‐radiometer (CS 2000, Konica Minolta Co., Ltd). The driving temperature of the wearable OLED was measured with a thermal imager (U5856A, Keysight Co., Ltd), and the operational lifetime was measured under a constant current condition through a Si Photodiode (Polaronix M9000S, McScience Co., LTD). The uniformity measurements based on the positions of the LED and OLED light sources were obtained using a laser power meter (Coherent Inc, FieldMaxII‐TO). The textile‐based OLED flexibility test was conducted using a bending test system (Science Town Inc.).

### Evaluation of Bilirubin Concentrations (In Vitro)

A newborn blood sample ready to be discarded after a general blood test at Eulji University Hospital was used. All samples were obtained with informed consent in advance. This evaluation was performed according to official guidelines and was approved by the Institutional Review Board (IRB Approval No. 2019‐04‐027‐005) of Eulji University. After the blood test, the remaining samples were separated and divided into six, and the bilirubin concentration of each sample was measured (0.16–0.17 mL). Bilirubin was measured by direct colorimetry (spectrophotometry) using a bilirubin meter B‐105 (Erma Inc, Yoshikawa‐shi, Saitama, Japan). Total bilirubin was measured and a tungsten lamp (12 V, 23 W) was used as the light source used for the measurement. The wavelengths used for the measurement were 455 and 575 nm and were measured with a photo cell detector. Neonatal blood from the capillary tube was centrifuged and mounted on a bilirubinometer to obtain results. Two samples were irradiated with light for 24 h with a blue light OLED having a 470 nm peak, two samples were irradiated for 24 h with a LED light of the same wavelength, and the remaining two samples were stored in a dark room environment. Then, bilirubin concentrations in the experimental group and the non‐irradiated group (control group) were measured.

### Statistical Analysis

Experimental results for bilirubin concentrations in the neonatal serum are expressed as mean ± standard deviation. There were 23 samples in the experimental results used in statistical analyses and they were analyzed by two‐tailed validation. For all tests, * means the *p*‐value is less than 0.001.

## Conflict of Interest

The authors declare no conflict of interest.

## Supporting information

Supporting Information

## Data Availability

The data that support the findings of this study are available from the corresponding author upon reasonable request.
