# Supplementary material for: Wearable Photomedicine for Neonatal Jaundice Treatment Using Blue Organic Light‐Emitting Diodes (OLEDs): Toward Textile‐Based Wearable Phototherapeutics
Source: Adv Sci (Weinh). 2022 Oct 30;9(35):2204622. doi: 10.1002/advs.202204622 (PMC9762290; doi:10.1002/advs.202204622)
Supplement: Supplementary file 1 — Supporting Information [file ADVS-9-2204622-s001.pdf]

ADVANCED SCIENCE
Open Access

Supporting Information

for *Adv. Sci.*, DOI 10.1002/advs.202204622

Wearable Photomedicine for Neonatal Jaundice Treatment Using Blue Organic Light-Emitting Diodes (OLEDs): Toward Textile-Based Wearable Phototherapeutics

Seungyeop Choi, Yongmin Jeon, Jeong Hyun Kwon, Chunhwa Ihm, Seung Yeon Kim* and Kyung Cheol Choi**

Supporting Information

Wearable Photomedicine for Neonatal Jaundice Treatment using Blue Organic Light-Emitting Diodes (OLEDs): Toward Textile-based Wearable Phototherapeutics

Seungyeop Choi[1†], Yongmin Jeon[2†], Jeong Hyun Kwon[5], Chunhwa Ihm[4], Seung Yeon Kim[3*], Kyung Cheol Choi[1*]*

[1]School of Electrical Engineering, Korea Advanced Institute of Science and Technology (KAIST), Daejeon 34141, Republic of Korea.
[2]Department of Biomedical Engineering, Gachon University, Seongnam 13120, Republic of Korea. [3]Department of Pediatrics, Nowon Eulji Medical Center, Eulji University School of Medicine, Seoul, Republic of Korea
[4]Department of Laboratory Medicine, Daejeon Eulji Medical Center, Eulji University School of Medicine, Daejeon, Republic of Korea
[5]Department of Display and Semiconductor Engineering, SUN MOON University, Choongcheongnam-do, Asan, 31460 Republic of Korea

[†] These authors contributed equally to this work.
[*]E-mail: kyungcc@kaist.ac.kr (Kyung Cheol Choi), dunggiduk@eulji.ac.kr (Seung Yeon Kim), haneul@eulji.ac.kr (Chunhwa Ihm)

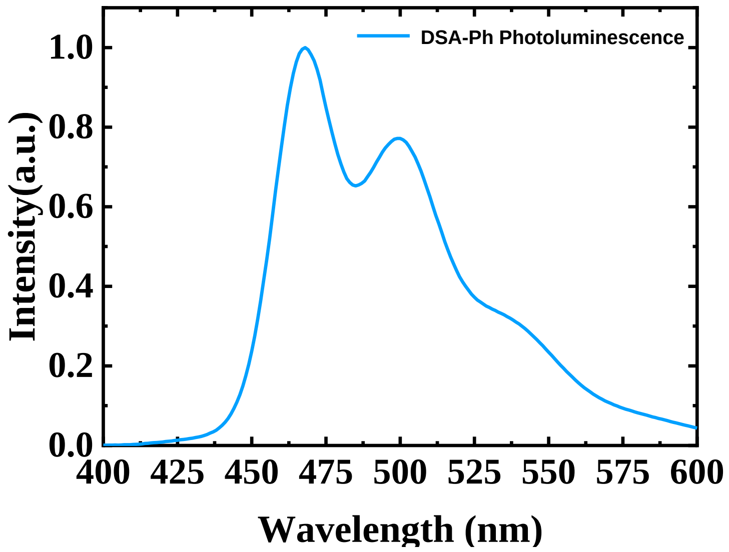

Figure S1. Photoluminescence graph of DSA-Ph selected as the Blue OLED EML material.

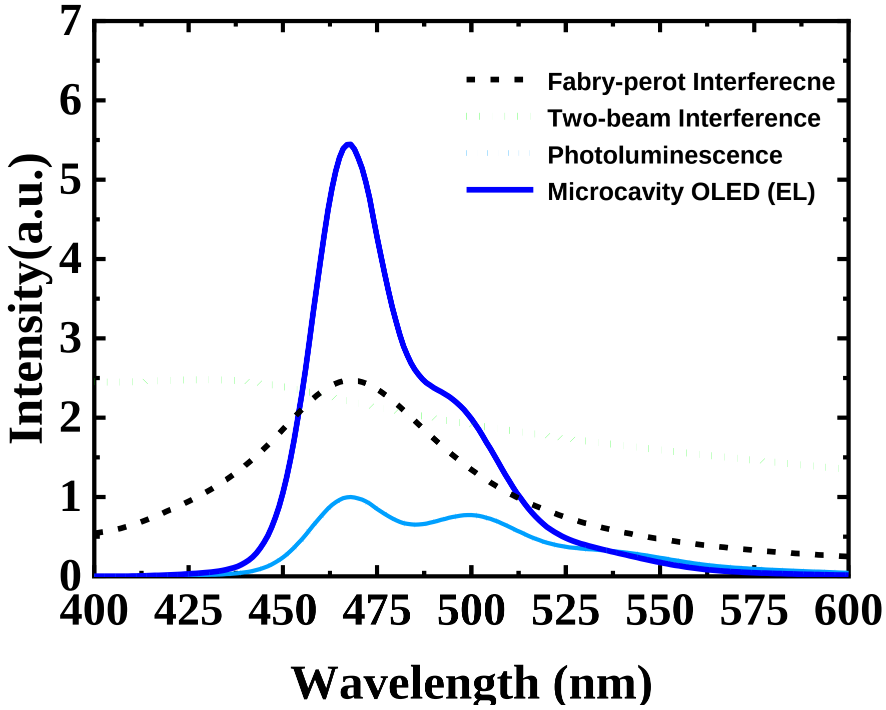

Figure S2. Wavelength optimization of blue OLED in the microcavity simulation

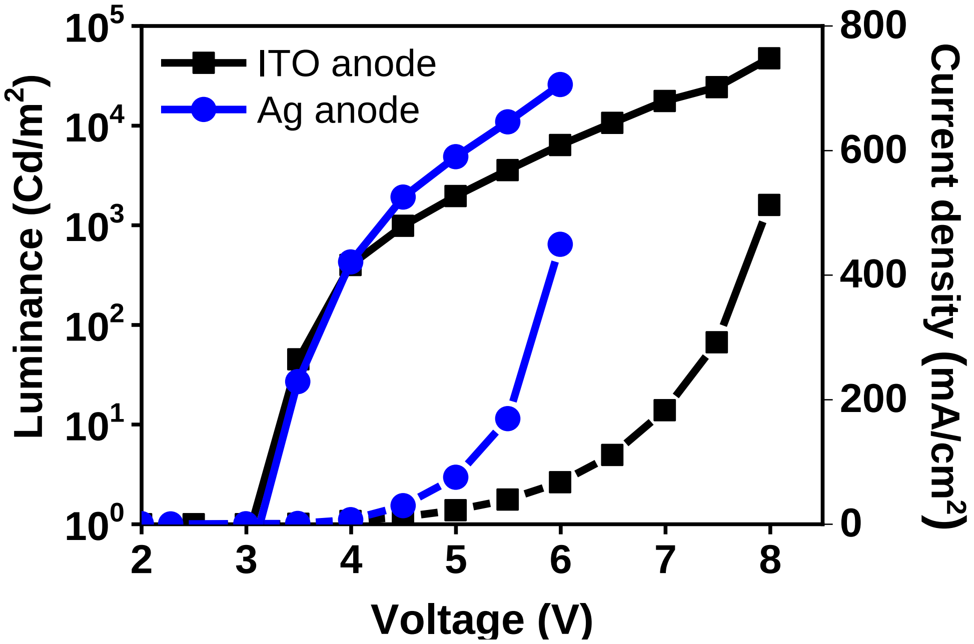

Figure S3. Luminance and current density graph according to OLED driving voltage

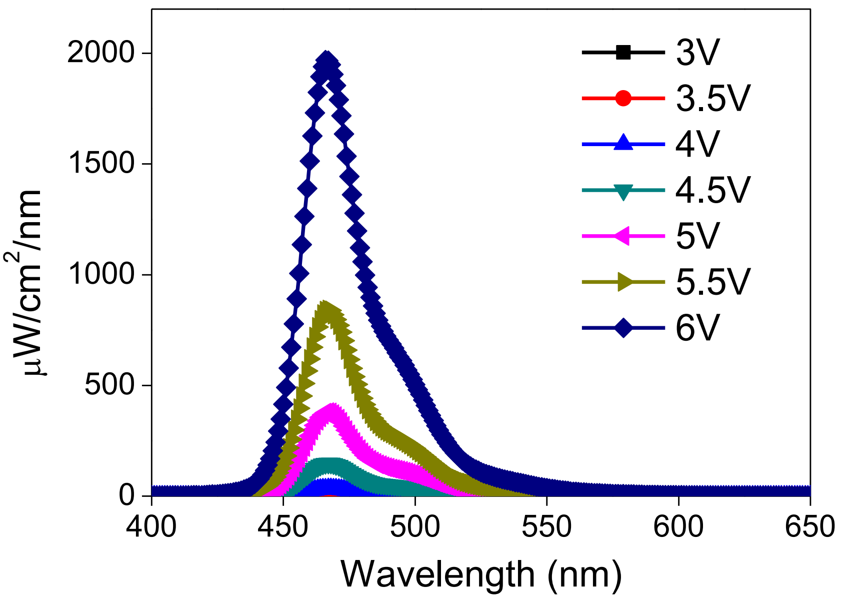

Figure S4. Emission intensity of textile-based blue OLEDs by wavelength and voltage

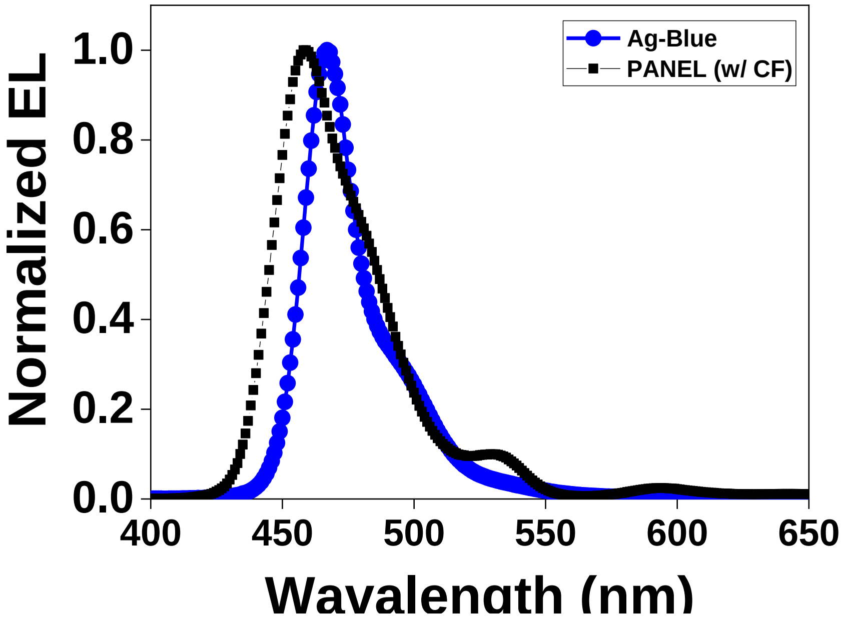

Figure S5. Comparison of wavelengths between textile-based blue OLED and large-area blue OLED.

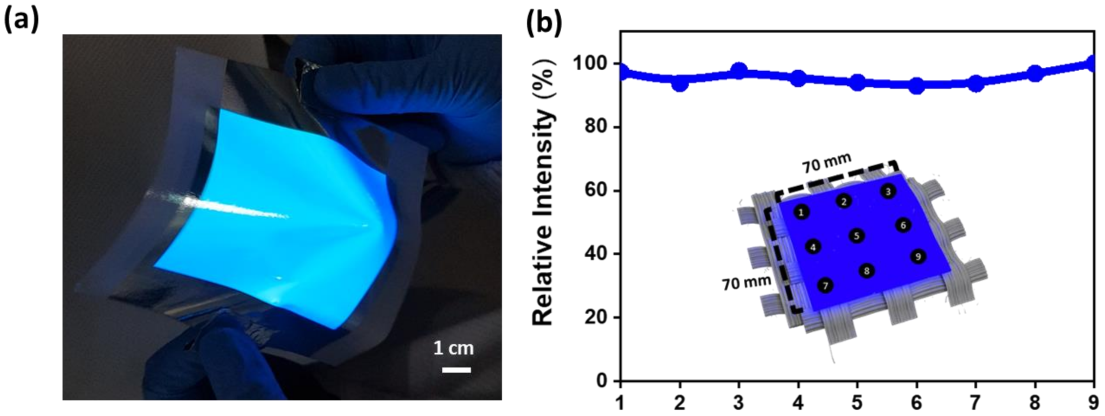

Figure S6. Uniformity test of wearable blue OLED intensity: (a) Large-area (70 mm × 70 mm) textile-based blue OLED **(b)** The 9-point uniformity of the OLED light source
